# Susceptibility of *Aedes aegypti* populations to pyriproxyfen in the Federal District of Brazil

**DOI:** 10.1590/0037-8682-0489-2019

**Published:** 2020-02-07

**Authors:** Bruno Lopes Carvalho, Rayssa Nádia Leite Germano, Kátia Maria Leal Braga, Evaldo Rosano Ferreira de Araújo, Douglas de Almeida Rocha, Marcos Takashi Obara

**Affiliations:** 1 Universidade de Brasília, Programa de Pós-Graduação Stricto Sensu em Medicina Tropical, Brasília, DF, Brasil.; 2 Ministério da Saúde, Secretaria de Vigilância em Saúde, Laboratório de Entomologia Médica, Brasília, DF, Brasil.; 3 Universidade de Brasília, Laboratório de Parasitologia e Biologia de Vetores, Programa de Pós-Graduação Stricto Sensu em Medicina Tropical, Brasília, DF, Brasil.

**Keywords:** Aedes aegypti, Susceptibility, Resistance, Pyriproxyfen

## Abstract

**INTRODUCTION:**

In Brasilia, pyriproxyfen (PPF; 0.01 mg/L) has been used for the larval control of *Aedes aegypti* mosquitoes since 2016. Information on the susceptibility of *Ae. aegypti* to PPF, and the development of resistance in populations from the Federal District of Brazil (FD) is limited. It is essential to monitor the susceptibility of *Ae. aegypti* to insecticides in order to improve vector control strategies. This study aimed to evaluate the susceptibility of *Ae. aegypti* populations from five areas of Brasilia to PPF.

**METHODS:**

We performed dose-response tests to estimate the emergence inhibition and resistance ratio of each field population, including the *Rockefeller* reference population. We also analyzed egg positivity, and the density and mortality of larvae and pupae.

**RESULTS:**

Populations from Vila Planalto (RR_50_=1.7), Regiment Guards Cavalry (RR_50_=2.5), and Sub-secretary of Justice Complex (RR_50_=3.7) presented high susceptibility to PPF, while the RR values of populations from Lago Norte (RR_50_=7.7) and Varjão (RR_50_=5.9) were moderately high, suggesting the emergence of insipient resistance to PPF in Brasilia. At 30 ng/mL, the highest larvae mortality rate was 2.7% for the population from Lago Norte, while that of pupae was 92.1% for Varjão and Vila Planalto.

**CONCLUSIONS:**

The five populations of *Ae. aegypti* from the FD are susceptible to PPF and there is a need to monitor the susceptibility of *Ae. aegypti* in new areas of the FD.

## INTRODUCTION

Arboviruses (Arthropod-borne viruses) are of great importance for public health due to their high impact on health and the economy^1^ ^,^ ^2^ ^,^ ^3^. The main viruses transmitted by mosquitoes are urban yellow fever (YFV), dengue (DENV), chikungunya (CHINKV), and Zika (ZIKV). Some researchers^4^ have reported the occurrence of at least one of these viruses in 146 countries, for which the main vectors are mosquitoes *Aedes aegypti* (Linnaeus, 1762) and *Aedes albopictus* (Skuse, 1894). 

*Ae. aegypti* is the primary vector of arboviruses in Brazil and is spread in all Federative Units of the country^5^. Environmental, socioeconomic, biological, and non-biological factors favor the dispersion and proliferation of this species, in addition to its urban habits, which are associated with anthropophilia, endophilia, endophagia, domiciliation, and oviposition strategy in artificial breeding sites. This increases the transmission of arboviruses^6^ ^,^ ^7^ ^,^ ^8^ ^,^ ^9^ ^,^ ^10^ ^,^ ^11^.

Historically, *Ae. aegypti* was controlled with organochlorine dichlorodiphenyltrichlorethane (DDT), organophosphates (malathion, fenitrothion), carbamate (bendiocarb), pyrethroids (cypermethrin, deltamethrin, and alphacypermethrin), biological insecticides (*Bacillus thunrigiensis*), and growth-regulating insecticides (diflubenzuron, novaluron, and pyriproxyfen)^5^.

The continuous and systematic use of the same product over a long period can select resistant individuals, compromising vector-control. Currently, resistance to organophosphates and pyrethroids has been reported in several populations of *Ae. aegypti*, including populations in the Federal District of Brazil (FD)^12^ ^,^ ^13^ ^,^ ^14^ ^,^ ^15^ ^,^ ^16^ ^,^ ^17^. 

In 1999, the National Network for Monitoring the Resistance of *Ae. aegypti* to Insecticides (MoReNAa) began to monitor the insecticide resistance of *Ae. aegypti* in Brazil, leading to changes in the products used in the National Program for Dengue Control (PNCD)^18^.

Temephos has been gradually replaced by diflubenzuron and novaluron since 2009. After this, the use of juvenile hormone analog pyriproxyfen (PPF) started in several Brazilian cities. In 2012, *Ae. aegypti* populations from Planaltina/FD were resistant to temephos and less susceptible to PPF, suggesting cross-resistance between Temephos and PPF^19^. 

In the FD, large-scale use of PPF began in 2016^20^ ^,^ ^21^. After 4 years, there is little information on the susceptibility profile of *Ae. aegypti* to PPF. This information is critical for improving control activities of *Ae. aegypti*. Thus, the objective of this study was to analyze the susceptibility of *Ae. aegypti* populations from five areas of the FD to PPF.

## METHODS

### Areas of study

The *Ae. aegypti* populations were derived from five areas of Brasilia, located in the Center-West region of Brazil. We established the selection criteria for the areas based on the use of PPF, during the last 3 years, carried out by the Environmental Surveillance Directorate (DIVAL). Thus, the selected areas were: i) Vila Planalto (15°47'33.3"S 47°50'56.6"W); ii) 1^st^ Regiment Guards Cavalry (RGC) located in the Urban Military Sector (15°45'37.4"S 47°57'16.8"W); iii) Lago Norte (15°44'11.0"S 47°51'36.8"W); iv) Varjão (15°42'30.7"S 47°52'45.4"W), and v) Sub-secretary of Justice Complex of the Federal District (SUAG-DF) (15°46'34.0 "S 47°56'26.9 "W), located in the Industry and Supply Sector.

### Field populations

We installed 60 ovitraps^22^, with the addition of 10% hay to increase egg capture yield^23^. All traps remained in each area for 2 weeks in the peridomicile environment. The traps were installed in the grounds of houses, protected from rain, with limited human and animal movement^24^. A volume of 20 mL of feno solution (10%) was added per trap to attract gravid females. The traps were replaced at the end of the first week and collected in the second week. The pallets containing eggs were collected, identified, and stored vertically inside a polystyrene box to prevent the eggs from being crushed or damaged. We transported the boxes to the Laboratório de Entomologia Médica, Secretaria de Vigilância em Saúde, Ministério da Saúde/SVS/MS. Then, we identified^25^ and counted eggs, which were classified as hatchlings, withered, and viable^26^, and estimated egg positivity index (EPI) and egg density (EDI). Pallets with eggs were submerged in dechlorinated water and hatched larvae were transferred to basins containing 1 L of dechlorinated water. Larvae were fed with 3 mg of natural Guabi® shredded cat food, which was added every 3 days. After the emergence of adults, we offered a 10% sugar solution to males and females. 

Three-days after their emergence, mosquitoes were fed with bird blood (*Gallus gallus domesticus*) every 48 h, according to Protocol No. 85/2018 of the Commission for Ethics in Animal Use (CEUA) of the University of Brasilia. Insectaries were maintained under controlled temperature (27 ± 2°C) and humidity (70 ± 15%) conditions. We used F1, F2, or F3 *Ae. aegypti* generations for the trials, according to the guidelines of the World Health Organization (WHO)^27^.

### Susceptible population

We used the *Rockefeller* population of *Ae. aegypti*, from the Laboratorio de Entomologia/Diretoria de Vigilância Ambiental/DIVAL/SES/DF.

### Chemical product

We used 97% technical grade Pyriproxyfen (PPF) provided by ROGAMA NEOGENV®.

### Biological tests

Biological bioassays were performed according as previously described^27^, using nine concentrations ranging from 0.001 to 30 ng/mL. For each dose, a total of 270 third-stage larvae were exposed, including the control group. Larvae were selected homogeneously to standardize their physiological and chronological age. Then, the larvae were placed in 400 mL cups containing 250 mL of distilled water, covered with a fine mesh net attached to the edge with an elastic alloy. All larvae remained at rest for approximately 30 min for acclimatization. Subsequently, we removed 1 mL of water from each beaker. Then, 1 ml of PPF solution was added in nine increasing concentrations and the mixture was homogenized with a glass rod. We fed the larvae with Guabi® Natural Feed every 72 h. Mortality was recorded every 48 h by a single researcher using a specific form; we completed this work when all pupae had emerged into adults. The mortality criteria were as follows: i) larvae and pupae unable to ascend to the surface or show diving reactions when the water was disturbed; ii) immobile larvae and pupae when stimulated with a needle in their siphon or cervical region ^27^ ^,^ ^28^ and, iii) adults that did not complete development and were unable to completely emerge from the pupa during the emergence phase. Live adults were considered as those totally free of their exuviae and able to fly or walk when gently touched. We performed all trials in triplicate on four different days and prepared an equal number of controls with the same amount of water and 1 mL of alcohol. Mortality, as well as the emergence of adults, was recorded when all the specimens in the control condition had emerged as adults. We discarded assays in which adult emergence was less than 90% in the control group. When inhibition was between 91 and 99%, the Abbott formula was used for correction^27^. We controlled temperature (25-30°C) and relative air humidity (70-80%) with a heater and a conventional air humidifier.

### Statistical analysis

We used the Polo PC program (Polo-PC, LeOra Software, Berkeley, CA)^29^(Raymond 1985)^30^to estimate the emergence inhibition doses of adults from the reference and field lines. The resistance ratios (RR) were determined through the EI_50_ quotient of the field population by the EI_50_ of the susceptible population, as well as the 95% confidence interval (CI 95%) of each population^31^. We also estimated the mortality of larvae and pupae^32^ ^,^ ^33^. The angular coefficient of the dose-response curve was calculated for each population using Graph-Pad Prism version 6.1 for Windows^34^. The criterion adopted for resistance classification was RR <5, indicating a susceptible field population; an RR between 5 and 10 indicated moderate resistance; and an RR >10 indicated high resistance^27^.

## RESULTS

**FIGURE 1:**
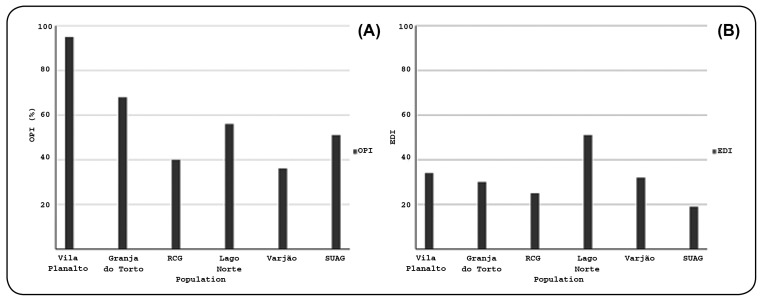
Ovitrap positive index (OPI) and egg density index (EDI) per positive trap in five areas of the Federal District of Brazil, from January to April 2017. **(A)** OPI: ovitrap positivity index, obtained by the percentage of positive paddles; and **(B)** EDI: egg density index obtained by the ratio between egg number and positive paddles.

The ovitraps obtained 5,966 eggs from *Ae. aegypti*, of which 4,171 were viable, 1,212 withered, and 583 hatched. The Figure 1 shows the OPI and EDI of the traps installed to obtain *Ae. aegypti* eggs. The highest OPI (95%) was recorded for the traps deployed in Vila Planalto, while the lowest values were recorded for those deployed in Varjão, whose OPI was 36%. Although Vila Planalto presented the highest OPI, the EDI was low (34). 

We exposed a total of 14,580 *Ae. aegypti* larvae to PPF. The Lago Norte and Varjão *Ae. aegypti* populations presented moderate resistance, with RR_50_ values of 7.7 and 5.9, respectively. The populations from Vila Planalto (RR_50_=1.7), RCG (RR_50_=2.5), and SUAG (RR_50_=3.7) presented high susceptibility to PPF, as shown in Table 1. 

**TABLE 1:** Estimates of inhibition emergence 50% (IE50) and resistance ratio of *Aedes aegypti* mosquito populations exposed to different doses of juvenile pyriproxyfen (PPF) hormone analog in 2018.

Population	Generation	EI50%(CI95%)	Slope	RR50%
Rockfeller		0.059 (0.005-0.199)	0.576	
Lago Norte	F1	0.56 (0.083-1.848)	0.546	7.7
Varjão	F1-F2	0.353 (0.043-1.388)	0.541	5.9
SUAG	F1-F2-F3	0.219 (0.007-1.055)	0.501	3.7
RCG	F1-F2	0.151 (0.002-0.772)	0.35	2.5
Vila Planalto	F1-F2-F3	0.106 (0.015-0.311)	0.543	1.7

Confidence Interval 95%. **EI:** emergence inhibition; **RCG:** 1^st^ Regiment Guards Cavalry; **SUAG:** Sub-secretary of Justice Complex of the Federal Distritct; **RR:** resistance ratio.

Table 2 presents data on the *Rockefeller* reference population, which obtained the highest EI compared to other field populations; thus, at a 30 ng/mL dose, the researchers recorded an average 99% EI of adults in the *Rockefeller* reference lineage. At this dose, the mean EI of adults in the field populations of *Ae. aegypti* was 92% for Vila Planalto and Varjão, 90% for Lago Norte, 89% for SUAG, and 87% for RCG.

**TABLE 2:** Emergence inhibition of *Aedes aegypti* populations exposed to different doses of juvenile pyriproxyfen (PPF) hormone analog in 2018.

Doses (ng/ml)	Rockefeller		Lago Norte		Varjão		SUAG		RCG		Vila Planalto	
	n (2,430)	*EI	n (2,430)	*EI	n (2,430)	*EI	n (2,430)	*EI	n (2,430)	*EI	n (2,430)	*EI
0.001	270	24	270	12	270	14	270	12	270	17	270	16
0.01	270	51	270	38	270	44	270	33	270	58	270	57
0.05	270	57	270	41	270	51	270	48	270	60	270	64
0.07	270	66	270	46	270	57	270	55	270	65	270	67
1	270	76	270	52	270	61	270	58	270	67	270	71
5	270	82	270	58	270	65	270	69	270	71	270	76
7	270	87	270	74	270	81	270	69	270	78	270	82
10	270	89	270	81	270	80	270	82	270	79	270	91
30	270	99	270	90	270	92	270	89	270	87	270	92

*EI: emergence inhibition (%).

**FIGURE 2:**
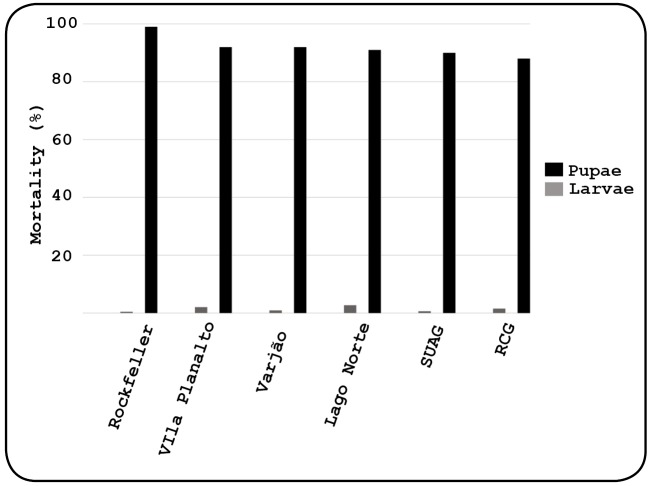
Mortality distribution of *Aedes aegypti* larvae and pupae from populations exposed to different doses of pyriproxyfen in 2018.

**FIGURE 3:**
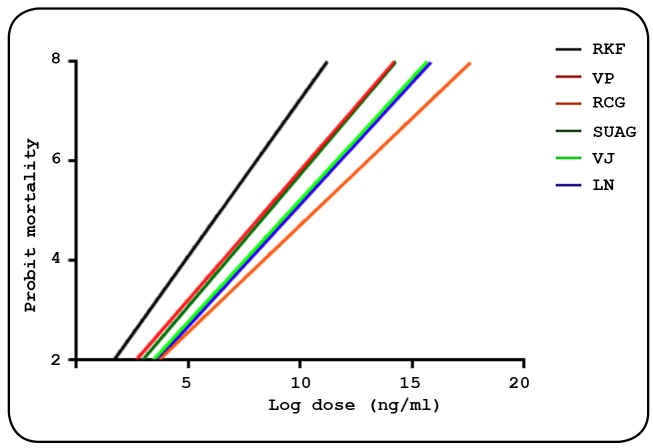
Mortality of *Aedes aegypti* populations on a logarithmic scale, in response to juvenile pyriproxyfen hormone analog in 2018. **RKF:** *Rockefeller*; **VP:** Vila Planalto; **RCG:** 1° Cavalry Regiment Guards; **SUAG:** Complex of the Undersecretary of Justice of the Federal District; **VJ:** Varjão; **LN:** Lago Norte.

Figure 2 shows the mortality rates of larvae and pupae. The mortality of larvae exposed to PPF was low, while it was high for pupae, with values above 90% in most field populations. The gradient values of the *Ae. aegypti* populations from five areas of the FD are shown in Figure 3. We observed gradient patterns similar to the reference population in those from Vila Planalto and SUAG. Although the *Ae. aegypti* populations from Varjão and Lago Norte were similar, when compared to the reference population, the RCG population showed less homogeneity. 

## DISCUSSION

Here, we evaluated the susceptibility of *Ae. aegypti* from the Federal District to the PPF. The results found for Vila Planalto (RR_50_=1.7), RCG (RR_50_=2.5) and SUAG (RR_50_=3.7) corroborate those reported by Leyva *et al.* (2010), who conducted technical PPF assays (97%) on four *Ae. aegypti* populations from Cuba. In that study, the RR values were 3.4, 0.9, 0.5, and 1 for populations of SANtem F13, Boyeros, Cotorro, and 10 de Octubre, respectively. 

Low levels of resistance were detected in two populations of *Ae. aegypti* from Barreiras (in the state of Bahia/BA [RR=1.4], and Bauru/SP [RR=3.6] following exposure to PPF, classifying them as susceptible to PPF^35^. In Martinique, *Ae. aegypti* populations also presented RR=2.2 in trials with 98.7% technical PPF^36^. Four-years later, Marcombe and collaborators detected susceptibility to PPF in eight populations of *Ae. albopictus* in the United States, obtaining RR values ranging from 1 to 2.36^37^. Despite the low RR values, periodic and systematic monitoring of *Ae. aegypti* populations over time in response to PPF is essential.

 Dose-response tests with PPF revealed that 30 ng/mL inhibited the emergence of adults by 99% (EI_99_) in the *Rockefeller* line; therefore, the diagnostic dose (DD=EI_99x2_) was estimated to be 60 ng/mL. No diagnostic-dose laboratory tests were performed; however, had they been conducted, the populations of Lago Norte (RR_50_=7.7) and Varjão (RR_50_=5.9) would have been considered susceptible, since they are likely to have inhibited 100% emergence. However, the moderately high values of RR_50_ indicated a probable change in susceptibility of *Ae. aegypti* populations, suggesting the emergence of resistance populations in Brasilia.

In Brazil, monitoring the insecticide resistance of *Ae. aegypti* populations has had an important impact on arboviruses epidemiology. Populations of *Ae. aegypti* with high levels of resistance contribute to the emergence of dengue outbreaks with high magnitude. In Campo Grande, the highest RR values (above 50 for deltamethrin) revealed that the period with the highest incidence of dengue coincides with the detection of *Ae. aegypti* populations with high resistance^38^.

Large urban centers with a greater flow of people, a history of mosquitoes and arbovirus circulation, are also determining factors for the increased spread of resistance. In 2019, the municipality of Palmas (RR_50_=28) had the highest probable number of dengue cases, unlike Caseara (RR_50_=1.6), which is less urbanized and is remote of other major centers urban areas^17^.

Thus, the monitoring of insecticide resistance in *Ae. aegypti* populations should be continuous and periodic for the rational management of adulticides and larvicides used to control mosquito populations, and for reducing local or large-scale resistance.

In this study, the mortality of larvae from field populations ranged from 0.6 to 2.0%, and that of pupae ranged from 99 to 88%, at a 30 ng/mL dose. This can be explained by the activity of PPF during the pupal phase, when comparative studies of field simulations between the *Rockefeller* and Itabuna/Bahia populations are conducted, with mortality rates of 97.9 and 95.1%, respectively^39^. Conversely, the larval mortality rate o was only 2.1 and 4.9%, respectively. Others studies^40^ have reported similar results under laboratory conditions, with higher mortality in *Ae. aegypti* pupae.

The mortality rate of *Ae. aegypti* pupae treated with PPF was 100% with the 0.2 and 1 ppm doses^41^. Another study using commercial PPF showed that doses lower than 0.01 ppb resulted in 98.5% pupal mortality^42^. Therefore, PPF is highly effective at inhibiting the emergence of adults, hindering the formation of wings, and the development of reproductive organs and external genitalia^43^.

Currently, the Brazilian Ministry of Health^44^uses the Larval Index Rapid Assay for *Aedes aegypti* (LIRAa) to direct actions for the control of *Ae. aegypti*, based on the detection of mosquito larval foci. Thus, breeding sites treated with PPF, in which the larvae remain alive, may lead to incorrect estimates of mosquito infestation levels, as well as the possibility of overlapping treatment of *Ae. aegypti* larval foci.

The limitations of this study included delay in obtaining the F1 generation of all populations, due to the very cold weather in 2017, which affected estimates of the dose-response curve. No new assays could be performed with PPF sub-doses to improve standardization of the diagnostic dose curve assays. 

New areas of the FD need to be monitored for changes in the susceptibility of *Ae. aegypti*. Field bioassays with *Ae. aegypti* populations from DF will also contribute to understanding the effectiveness of PPF in the field.
